# In vivo and in vitro investigation of skin anti-inflammatory effect of Sumac (*Rhus coriaria* L.)

**DOI:** 10.1007/s10787-026-02368-2

**Published:** 2026-08-26

**Authors:** Marco Fumagalli, Giulia Martinelli, Silvio Sosa, Zixiong Tang, Michela Carlin, Safwa Moheb El Haddad, Nicole Maranta, Carola Pozzoli, Chiara Di Lorenzo, Giovanna Baron, Stefano Piazza, Marco Pelin, Mario Dell’Agli, Enrico Sangiovanni

**Affiliations:** 1 Department of Pharmacological and Biomolecular Sciences “Rodolfo Paoletti”, Università degli Studi di Milano, 20133 Milan, Italy; 2 Department of Life Sciences, University of Trieste, 34127 Trieste, Italy; 3 Department of Pharmaceutical Sciences, Università degli Studi di Milano, 20133 Milan, Italy

**Keywords:** *Rhus coriaria*, Skin inflammation, In vivo, Keratinocyte

## Abstract

**Background:**

Sumac (*Rhus coriaria* L., Anacardiaceae) fruit is receiving a growing attention as potential nutraceutical and cosmetic ingredient. It is appreciated either as a spice and medicinal remedy in Middle East countries. The application of fruits preparations to skin injuries emerged from ethnopharmacological studies, sustained by few experimental studies on wound healing. The present work, based on previous evidence of in vitro anti-inflammatory activity in human keratinocytes (HaCaT), is aimed at translating the in vitro effect of sumac to an in vivo model of dermatitis.

**Results:**

Two polar sumac fruit extracts (acetone extract, ARC; ethanol extracts, mERC) were investigated in mice by Croton oil-induced ear dermatitis model. The in vivo topical anti-inflammatory activity was investigated 6 h and 24 h after dermatitis induction by means of edema, leukocyte infiltrate, and histological features; the expression of inflammatory genes was also analyzed by PCR array. Biological targets identified in vivo were validated in vitro by stimulating HaCaT cells with pro-inflammatory mediators for the same exposure time-points (6 h and 24 h). Both the extracts inhibited PMA-induced IL8 release in HaCaT cells, but mERC showed lower IC_50_ (18.68 μg/mL vs. 26.34 μg/mL, respectively). According to the in vitro effect, both the extracts (300–1000 µg/mL) reduced edema and neutrophilic granulocytes infiltrate in mice auricle at 6 h and 24 h after dermatitis induction, with comparable activity. The gene expression profile in the inflamed ear tissues suggested the involvement of TNF and IL4/IL13 pathways in ARC and mERC anti-inflammatory activity. This hypothesis was confirmed by further in vitro experiments on HaCaT cells, in which both extracts impaired TNF-induced IL-8 with IC_50_ below 1.5 μg/mL. Once, again mERC showed slightly lower IC_50_ then ARC (1.32 μg/mL vs. 1.63 μg/mL). In analogy, the most promising extract also impaired the release of IL-4-induced CCL26, with IC_50_ of 1.06 µg/mL.

**Conclusions:**

This study supports the relevance of the biological properties of sumac fruit for skin inflammation. Moreover, mechanisms of action related to TNF and IL4/IL13 pathways suggest further specific investigations related to skin allergy and autoimmune diseases.

**Supplementary Information:**

The online version contains supplementary material available at https://doi.org/10.1007/s10787-026-02368-2.

## Background

Inflammatory skin diseases are the most common dermatological disorders, and their pathophysiology has been deeply investigated over the last few decades. The discovery of inflammatory biomarkers has led to the development of novel biological treatments, such as anti-TNF-α, anti-IL23, and anti-IL4 antibodies, showing groundbreaking results in severe diseases (Dubin et al. 2021; Singla et al. 2023). The need for safe and innovative treatments have also prompted research into compounds from natural sources and the creation of new synthetic small molecules (Butala et al. 2023; Roy et al. 2023).

The traditional use of medicinal plants still inspires dermatological pharmacological research, representing a valuable source of information for drug discovery (Fernandes et al. 2023). *Rhus coriaria* L. is a Mediterranean plant belonging to the Anacardiaceae family, commonly known as “sumac”. The popularity of sumac fruit is mainly due to its consumption as a sour spice typical of the Middle Eastern culinary tradition. Based on available ethnopharmacological data, several scientific studies have been carried out to investigate the biological properties and the phytochemical composition of sumac fruit, including ours (Nozza et al. 2020; Martinelli et al. 2022; Khalilpour et al. 2019). Specifically, the high concentration of polyphenols, such as flavonoids and gallotannins has been associated with hypoglycemic, anti-inflammatory, antioxidant, and antibacterial activities (for recent reviews on the topic, refer to Alsamri et al. (2021); Elagbar et al. 2020; Hashem-Dabaghian et al. 2022). Accordingly, sumac has long been used in Persian traditional medicine for wound healing, metabolic diseases, and gastrointestinal disorders (Said et al. 2002; Calabro et al. 2023; Rodriguez-Castillo et al. 2025). Furthermore, the plant has long-standing use for skin injuries including burns, wounds, and eczema in several Middle Eastern countries (Altundag and Ozturk 2011; Sezik et al. 1991). However, a few in vivo studies have suggested that the topical application of polar extracts from sumac fruit may contribute to wound healing through antibacterial and collagen-promoting activities (Alsarayreh et al. 2022; Gabr and Alghadir 2019).

We previously demonstrated that the traditional maceration of fruit in ethanol (mERC) yields a polyphenol-rich extract with a stronger in vitro anti-inflammatory effect in human skin keratinocytes (HaCaT) challenged with TNF-α, as compared to a water extract (Khalilpour et al. 2019). Moreover, we compared mERC with an acetone extract (ARC), preferentially rich in tannins, in a model of *H. pylori*-induced inflammation, showing comparable bioactivity (Martinelli et al. 2022).

To the best of our knowledge, the in vivo anti-inflammatory activity of sumac at the skin level still requires experimental evidence. To this end, we sought to translate our in vitro findings into in vivo evidence by evaluating the ability of *Rhus coriaria* extracts to inhibit the Croton oil-induced ear dermatitis in mice, after topical application. Croton oil, derived from *Croton tiglium* seeds contains phorbol myristate acetate (PMA), a protein kinase C activator that influences several cellular processes and can trigger an inflammatory response. This property can be exploited experimentally to reproduce a dermatitis in the skin (Griswold et al. 1998; Ivetic Tkalcevic et al. 2012). In addition, an in vitro approach using HaCaT skin keratinocytes exposed to PMA was applied to characterize the activity of the extracts on selected targets identified in the in vivo study.

## Methods

### Materials and animals

Croton oil, indomethacin, hydrocortisone, tetramethylbenzidine (TMB), sodium azide, and hexadecyltrimethylammonium bromide (HTAB) were purchased from Sigma-Aldrich (Milano, Italy). Ketamine hydrochloride (Ketavet 100) was purchased from MSD Animal Health (Segrate, Italy). Male Hsd:ICR (CD-1) mice (body weight: 28–32 g, 6–9 weeks old) were supplied by Envigo RMS (San Pietro al Natisone, Italy).

EGCG and apigenin were purchased from PhytoLab GmbH & Co. KG (Vestenbergsgreuth, Germany). Disposable materials for cell culture were from Primo®, Euroclone (Pero, Italy). Proinflammatory cytokines TNF-α and IL4 were from PeproTech (Thermo Fisher Scientific; Monza, Italy) (Merck Life Science; Milano, Italy), while PMA was from Merck Life Science (Milano, Italy).

### Plant material and extraction

*Rhus coriaria* L. fruit sample was collected by a local market in the Taleghan region located 120 km northwest of Teheran, Iran, as previously described (Khalilpour et al. 2019). The plant is not an endangered or protected species. The sample was authenticated by the Herbarium Unit of the School of Biological Sciences (University of Science Malaysia), where a voucher specimen was deposited (ref. #11,526).

Two different methods of extraction were included in this investigation, based on previous studies (Martinelli et al. 2022; Khalilpour et al. 2019): dried fruits were milled by an electric blade miller; then, to obtain the ethanol macerate (mERC), 5 g of plant material were extracted by soaking the powder with ethanol (50 mL) for 48 h at room temperature under gentle shaking by magnetic stirrer. Otherwise, the same quantity of powder was extracted by acetone (50 mL), based on a double extraction procedure, thus obtaining the acetone extract (ARC): in brief, fruits were extracted for 4 h, followed by 16 h of a second extraction on residual plant materials to obtain the fruit exhaustion.

Both solvents were removed by rotary evaporator (Laborota 4000, Heidolph Instruments GmbH & Co. KG, Schwabach, Germany) at 40 °C and reduced pressure. The resulting extracts were kept at 2 °C, while stock aliquots (50 mg/mL) for cell treatments prepared by DMSO solution were kept at − 20 °C. The extraction yields were measured: 22.08% (w/w) for ARC, and 21.60% (w/w) for mERC, respectively.

### Assessment of the in vivo anti-inflammatory activity

Experiments were carried out at the University of Trieste (Italy), in compliance with the Italian (Legislative Decree 26/2014) and European Union (EU Directive 2010/63/EU) law and policies; thus, all experiments were conducted in accordance with OECD guidelines. The experimental study was approved by the University Body for Animal Well-being (OPBA) of the University of Trieste and the Italian Ministry of Health (Decree 826/2021-PR of 28th October 2021).

#### Croton oil-induced ear dermatitis

A local dermatitis was induced in the right auricle of mice, as previously described (Tubaro et al. 1986). Male Hsd:ICR (CD-1) mice 6–9 weeks old, weighing 28–32 g, were anesthetized by intraperitoneal injection of ketamine hydrochloride (145 mg/kg) before the application of Croton oil (80 μg, dissolved in 15 μL acetone) to the inner surface of the right ear (surface: about 1 cm^2^). The left ear remained untreated, considering that the vehicle did not influence the inflammatory reaction, as previously observed. Control animals received only the irritant solution, whereas the others received both the irritant and the substances under test. After 6 h and 24 h from the dermatitis induction, at the peak of vascular (edema) and cellular (inflammatory cells infiltrate) phase of inflammation, respectively, mice were sacrificed by cervical dislocation. A punch (diameter: 6 mm) was taken from both the auricles to evaluate the edematous response, inflammatory cells infiltrate (mainly neutrophilic neutrophiles) and the expression of selected pro-inflammatory genes. Additionally, part of each auricle was fixed in 10% formalin for histological analysis. A total of 8 to 10 mice were used for each group of treatment.

#### Evaluation of the edematous response

In each mouse, edema was quantified as weight difference between the punches taken from the treated and untreated auricles. The anti-edema activity was expressed as percent inhibition of the edematous response in mice treated with the test substances in comparison to edema measured in mice treated with the irritant alone (controls).

#### Evaluation of the neutrophilic granulocyte infiltrate

The neutrophilic granulocyte infiltrate was quantified in the treated auricles (half punch of the same plug used to quantify edema), measuring the myeloperoxidase (MPO) activity, as index of neutrophilic granulocytes presence (Giangaspero et al. 2009). MPO was extracted by HTAB, according to Bradley et al. (Bradley et al. 1982), and the enzyme activity was measured by a colorimetric assay using TMB as chromogen (Andrews and Krinsky 1981). Each auricle plug, suspended in 1 mL buffered saline (0.1 M sodium acetate buffer, pH 4.2), containing 0.1% HTAB (w/v), was homogenized by Ultra-Turrax (Ika-Werk; Staufen, Germany) for 5 s at 20,000 rpm. After homogenate centrifugation at 15,000 g for 5 min, MPO activity was measured in the supernatant. In each well of a 96-well microplate, 25 μL of the supernatant were mixed with 50 μL of the chromogen solution (2.83 mM TMB in 0.1 M sodium acetate buffer, pH 4.2, containing 0.1% HTAB), and MPO reaction was started by the addition of 75 μL of 0.7 mM hydrogen peroxide. After 5 min at 25 °C, the enzyme was blocked by 50 μL of 4 M acetic acid, containing 10 nM sodium azide. Then, absorbance was measured at 620 nm using an automated microplate reader (Bio-Tek Instruments, Winooski, VT, USA). MPO activity was expressed as enzyme units in 1 mL of supernatant. One unit of MPO was defined as the amount of enzyme oxidizing 1 nmol of TMB/min. MPO activity of each sample was determined in duplicate.

#### Histological analysis

Formalin-fixed auricle biopsies were dehydrated with ascending ethanol concentrations, cleared in xylene, and embedded in paraffin wax. Sections (20 μm) were stained with hematoxylin–eosin and observed using an inverted light Leica DMi1 microscope equipped with a FLEXACAM C1 standard camera (Leica Microsystems; Milano, Italy).

### PCR analysis

For PCR analysis, RNA was firstly isolated from subsamples of auricles (half punch of the same plugs used to quantify edema) stored at − 80 °C through NucleoSpin® RNA Plus extraction kit (Macherey–Nagel, Düren, Germany), according to manufacturer’s instructions. In brief, cells were homogenized and lysed by a sample buffer (350 μL) after 6 h of treatment. DNA was removed by exclusion columns, following several steps of washing and centrifugation. The RNA was bound onto another silica column and finally eluted by RNAase free water (60 μL). The amount of RNA and its quality were verified by spectrometry at 260/280 nm (NanoDrop ND‐1000, Thermo Fisher Scientific, Waltham, MA, USA).

The regulation of inflammatory markers was measured by a PCR RT^2^ Profiler™ (PAMM011ZE-4) for Mouse Inflammatory Cytokines & Receptors (QIAGEN, Hilden, Germany), including 84 target genes, 5 housekeeping genes, 3 positive PCR controls, and 3 reverse transcription controls.

The cDNA was synthetized by RT^2^ First Strand kit, starting by 400 ng of RNA sample, and used for PCR analysis, following the manufacturer's instructions (QIAGEN, Hilden, Germany). The cDNA was then mixed with the SYBR Green Master Mix RT^2^ reagent and loaded into the 384‐well profiler. Amplification and quantification steps were performed by C1000TM Thermal Cycler coupled with CFX384TM Real‐Time PCR Detection System (Bio‐Rad Laboratories, Segrate, Italy).

The raw Ct values were summarized in a template (.xls) use to create a table of values with cut-off set to 35, following the procedures defined by the manufacturer’s web portal SABiosciences (QIAGEN, Hilden, Germany). This table was uploaded onto the data analysis web portal at http://www.qiagen.com/geneglobe to obtain a final data report. Ct values were normalized based on a manual selection of the most reproducible housekeeping genes. The web platform calculated the Fold Change using delta delta Ct method: delta Ct is calculated between inflammatory genes of interest and an average of housekeeping genes. Thus, the Fold Change is calculated as 2^(− delta delta CT). The data analysis web portal also automatically generated scatter plots, heat maps, and raw data tables reporting the Fold Change values and its significance.

### Cell culture and treatment

HaCaT cells are spontaneously immortalized human keratinocytes (Cell Line Service GmbH; Eppelheim, Germany); they were cultured in 75 cm^2^ flasks for 72 h, under humidified atmosphere (5% CO^2^, 37 °C) in high-glucose DMEM (Merck Life Science, Milano, Italy). The subculture was obtained by EDTA 0.25% followed by EDTA-trypsin detachment (GibcoTM; Thermo Fisher Scientific, Monza, Italy). The culture media was supplemented with 100 units penicillin/mL, 100 mg streptomycin/mL, 2 mM L-glutamine (GibcoTM; Thermo Fisher Scientific, Monza, Italy), and 10% heat-inactivated fetal bovine serum (FBS) (Euroclone S.P.A., Milan, Italy).

For all experiments, cells were seeded in 24 well plates at a density of 60 × 10^3^ cells/well. After 72 h of growth in supplemented medium, treatments were performed for 6 h and 24 h in FBS-free medium. Plant extracts were diluted from stock aliquots (50 mg/mL) into cell medium added to TNF-α, IL4, or PMA. The pro-inflammatory stimuli were stored at − 80 °C until the day of treatment. PMA was dissolved into DMSO (1 mM stock solution), whereas cytokines were dissolved into sterile water (100 µg/mL stock solution). Decreasing concentration curves of extracts were obtained by serial dilution. EGCG and apigenin (20 µM) were dissolved DMSO at the concentration of 40 mM and 20 mM, respectively, and used as reference anti-inflammatory compounds(Martinelli et al. 2022; Piazza et al. 2022).

### MTT assay

The integrity of the cell morphology before and after each treatment was assessed via light microscope inspection. The toxicity of plant extracts was excluded by the 3,4,5-dimethylthiazol-2-yl-2-5-diphenylte-trazolium bromide method (MTT test), as previously described (Khalilpour et al. 2019). After the development of purple tetrazolium salt, cells were lysed by isopropanol/DMSO solution and the absorbance was acquired at 550 nm (VICTOR X3; PerkinElmer, Milano, Italy). Viability was expressed as a percentage (%) relative to untreated control, which was arbitrarily assigned the value of 100%. Values above 80% were considered acceptable for convention.

### ELISA assay

Human IL8 and CCL26 levels were measured by sandwich ELISA assay (PeproTech, Thermo Fisher Scientific, Monza, Italy), carried out on 100 µL of cell supernatants at the end of treatment. Supernatants were stored at −20 °C until the assay conducted as previously described following the manufacturer’s instructions (Martinelli et al. 2022; Khalilpour et al. 2019). In brief, EIA/RIA plates (Merck Life Science, Milano, Italy) were coated with anti-IL8 or anti-CCL26 coating antibody. The day after, the plate was blocked by an excess of BSA for 1 h; then, samples were loaded for 2 h to allow the link with the first antibody. The second biotinylated antibody was added for 2 h, HRP-avidin construct was formed, and a colorimetric reaction was started by the addition of ABTS substrate. The absorbance was read at 405 nm (VICTOR X3; PerkinElmer, Milano, Italy) and converted into pg/mL by using the calibration curve of each standard protein (0–1000 pg/mL). Data were expressed as a percentage (%) relative to the inflammatory condition (TNF-α, IL4, PMA) which was arbitrarily assigned the value of 100%.

### LC-HRMS conditions and data analysis

The mERC extract was analyzed at a concentration of 2 mg/mL (dissolved in the starting mobile phases composition) by LC-HRMS as described by Baron et al. (Baron et al. 2021), using a RP chromatographic separation followed by a LTQ Orbitrap XL mass analyzer as detector set to acquire both Full MS and MS/MS spectra. A targeted data analysis was performed by building a database containing compounds already known in the literature to be present in *Rhus coriaria* L. (entries n=282) (Abu-Reidah et al. 2015; Grassia et al. 2021; Mazzara et al. 2023; Tohma et al. 2019). Moreover, a calculation of possible high molecular weight gallotannins *m/z* was carried out to verify their presence as [M-2H]^2−^ and thus adding these compounds to the database. The annotation was obtained through the accurate mass (mass tolerance of 5 ppm), isotopic and fragmentation patterns.

### Statistical analysis

All biological data were expressed as mean ± SEM of at least three experiments (n=3). The statistical significance (*p* value ≤ 0.05) was determined using unpaired one‐way ANOVA analysis of variance followed by Bonferroni post hoc‐test (in vitro data) or Dunnett’s test (in vivo data), using GraphPad Prism 9.0 software (GraphPad Software, San Diego, CA, USA). Statistics concerning data from PCR array were analyzed by data analysis web portal at http://www.qiagen.com/geneglobe, as previously described (t-test).

## Results

### Inhibitory effect of sumac extracts against PMA-induced IL8 release in HaCaT cells

**Fig. 1:**
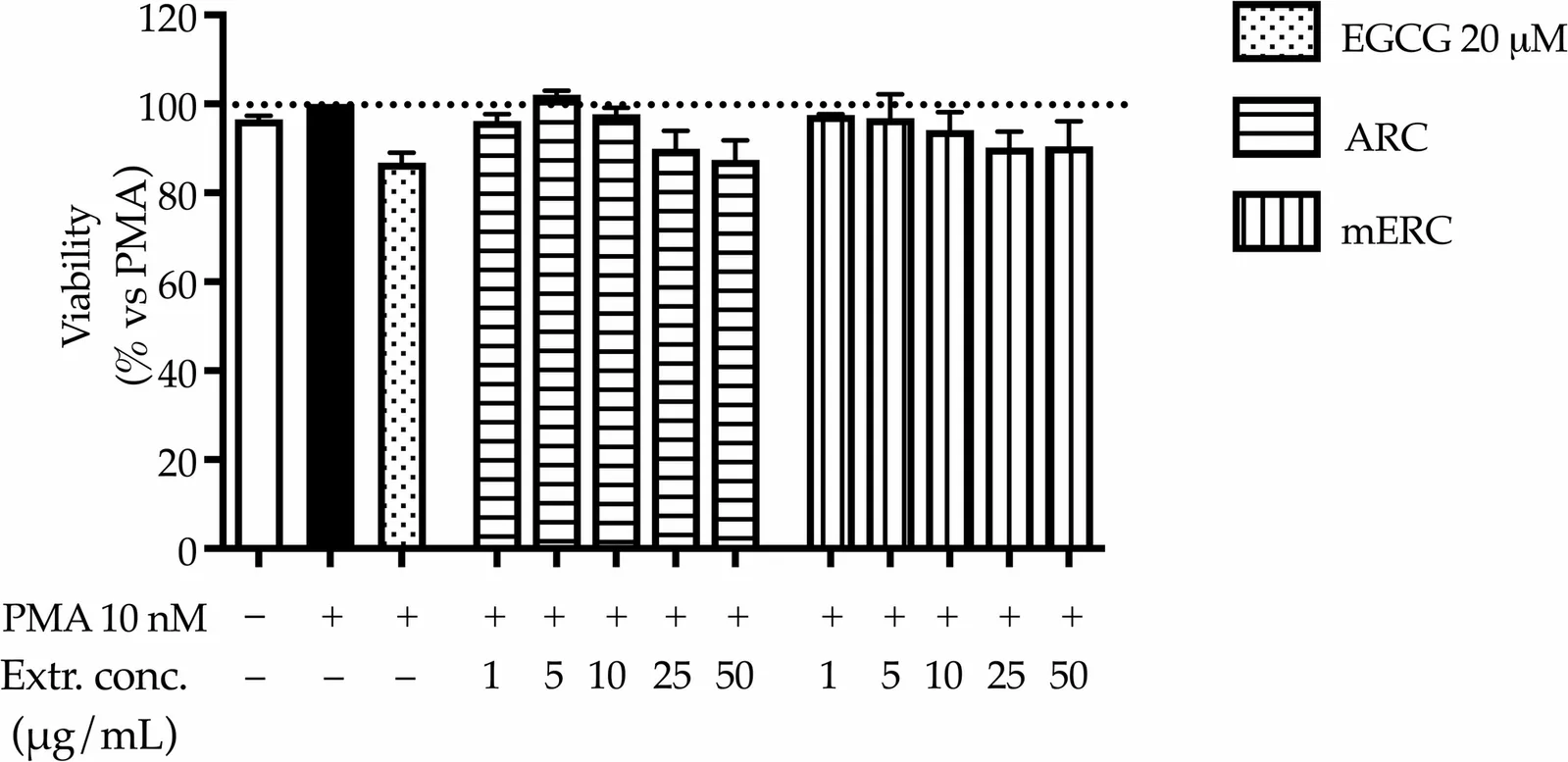
MTT assay on HaCaT cells exposed to mERC or ARC and PMA for 24 h. The data are expressed as percent of cell viability, relative to PMA stimulated control cells (black column), which is arbitrarily assigned a value of 100%. ARC, acetone extract from *Rhus coriaria* L. fruit; mERC, macerate ethanol extract *Rhus coriaria* L. fruit; EGCG, epigallocatechin gallate (20 μM)

Two types of sumac extracts (mERC and ARC) emerged as the most promising candidates for anti-inflammatory activity and safety profile (0–100 µg/mL) in human keratinocytes (HaCaT) and gastric epithelial cells (GES-1) in our previous work (Martinelli et al. 2022; Khalilpour et al. 2019). These extracts were further evaluated for their topical anti-inflammatory activity at the skin level. As a first step before in vivo translation, the cytotoxicity of sumac extracts (1–50 µg/mL) was excluded by MTT assay during PMA stimulation in HaCaT cells for 24 h (shown in Fig. 1), while cytotoxicity during TNF-α stimulation was previously ruled out (Martinelli et al. 2022; Khalilpour et al. 2019).

**Fig. 2:**
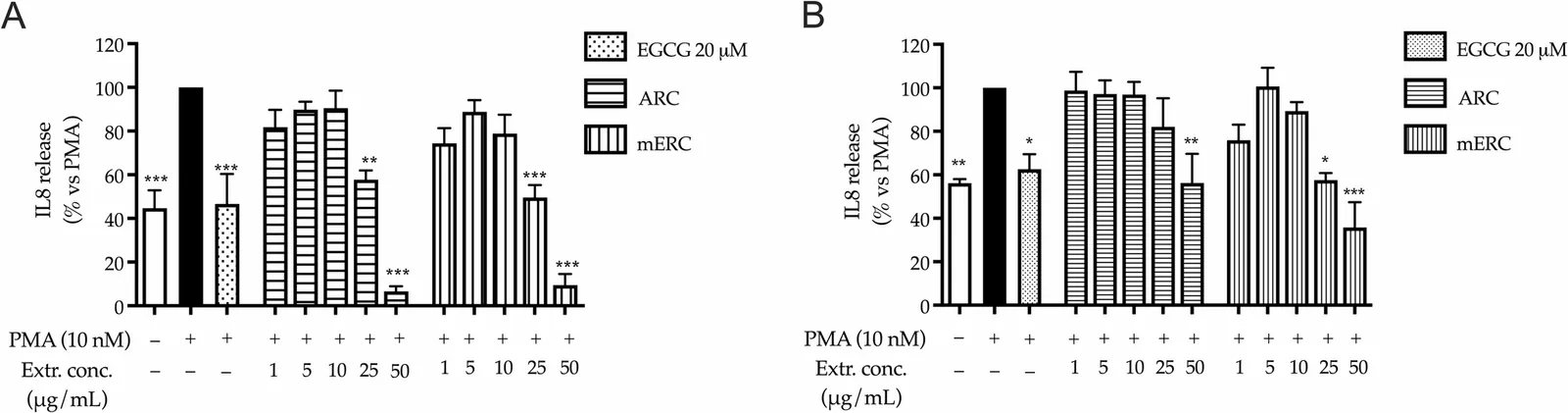
IL8 release by HaCaT cells exposed to mERC or ARC and PMA for 6 h (**A**) or 24 h (**B**). IL8 release was evaluated by the ELISA assay. Calculated IC_50_ at 24 h were: 26.34 μg/mL for ARC, and 18.68 μg/mL for mERC, respectively. The data are expressed as percent of IL8 release, relative to control cells exposed only to PMA (black column), which is arbitrarily assigned a value of 100%. ARC, acetone extract from *Rhus coriaria* L. fruit, mERC, macerate ethanol extract *Rhus coriaria* L. fruit. EGCG, epigallocatechin gallate (20 μM). *p < 0.05, ** *p* < 0.01, *** *p* < 0.001 (one-way ANOVA) versus control cells exposed only to PMA

Since no impact on cell viability was observed, subsequent experiments evaluated the ability of the extracts to inhibit IL8 release by HaCaT cells exposed to PMA for 6 or 24 h. IL8 was chosen as a significant skin inflammatory marker selected from previous studies (Martinelli et al. 2022; Khalilpour et al. 2019). Although both extracts inhibited IL8 release, mERC showed a more promising concentration–response trend compared with ARC, both after a 6 h and 24 h exposure (shown in Fig. 2A, B). At 24 h, the IC_50_ of mERC was 18.68 µg/mL (95% CI: 10.97–31.82 µg/mL), while the IC_50_ of ARC was 26.34 µg/mL (95% CI: 15.53–30.42 µg/mL). These promising results prompted the in vivo evaluation of the anti-inflammatory activity, specifically regarding the ability of the extract to inhibit the Croton oil-induced ear dermatitis after topical application.

### Anti-inflammatory effect of sumac extracts in Croton oil-induced dermatitis model

The in vivo topical anti-inflammatory activity was evaluated at two time points to observe early and late events: at 6 h after Croton oil-induced dermatitis (the time of maximum edema formation) and after 24 h (the time of the maximumneutrophilic granulocyte accumulation) (Giangaspero et al. 2009). The doses, ranging from 100 to 1000 µg/cm^2^, were selected based on in vitro measurements to scale the potential biological activity. In line with in vitro experiments, both extracts induced a dose-dependent edema reduction both 6 h and 24 h after dermatitis induction. In particular, 6 h after dermatitis induction, mERC exerted a significant effect (20% and 31% edema reduction) at the doses of 300 and 1000 µg/cm^2^. ARC induced a significant edema reduction (19%) even at the lowest dose, reaching up to a 46% reduction at 1000 µg/cm^2^. As expected, the reference steroidal and non-steroidal anti-inflammatory drugs, hydrocortisone (20 µg/cm^2^) and indomethacin (100 µg/cm^2^), induced 57% and 64% edema reduction, respectively. At 24 h, only the highest dose of mERC (1000 µg/cm^2^) was significantly effective (41% edema reduction), whereas the effect of ARC was significant (31% and 55% reduction) at the doses of 300 and 1000 µg/cm^2^. The reference drug indomethacin lost its anti-edema effect after 24 h, as previously reported (Tubaro et al. 1986), whereas hydrocortisone reduced edema formation by 52% (Table 1). These results demonstrate that the topical application of sumac extracts reduces the inflammatory vascular events underlying edema formation.

**Table 1:** Topical anti-inflammatory activity of *Rhus coriaria* extracts: effect on ear edema induced by Croton oil in mice

Group of treatment	N°. an	Dose (µg/cm^2^)	6 h	Inhibition (%)	24 h	
			Edema (mg) Mean ± SEM		Edema (mg) Mean ± SEM	Inhibition (%)
Control	10	–	7.0 ± 0.2	–	2.9 ± 0.2	–
mERC	10	100	6.4 ± 0.4	9	2.8 ± 0.2	3
	10	300	5.6 ± 0.3**	20	2.3 ± 0.3	21
	10	1000	4.8 ± 0.2**	31	1.7 ± 0.2**	41
ARC	10	100	5.7 ± 0.2**	19	2.7 ± 0.2	7
	10	300	4.6 ± 0.3**	34	2.0 ± 0.3*	31
	10	1000	3.8 ± 0.2**	46	1.3 ± 0.2**	55
Indomethacin	10	100	3.0 ± 0.3**	57	2.9 ± 0.4	0
Hydrocortisone	9	20	2.5 ± 0.2**	64	1.4 ± 0.1**	52

*p<0.05, **p<0.001 versus control at the analysis of variance followed by Dunnett’s test

Furthermore, the myeloperoxidase (MPO) activity was measured in the inflamed auricle as an index of neutrophilic granulocyte infiltration. Data reported in Table 2 clearly show that the sumac extracts (100–1000 μg/cm^2^) reduced also MPO activity both at 6 h and 24 h after dermatitis induction, in line with their effect on edema formation. At 6 h, the enzyme activity reduction induced by mERC was significant at 300 and 1000 μg/cm^2^ (23% and 29%), similarly to that induced by the same doses of ARC (28 and 36% reduction). After 24 h, only the highest dose of mERC (1000 μg/cm^2^) provoked a significant MPO activity reduction (39%), whereas the enzyme activity reduction induced by ARC was significant at doses of 300 and 1000 μg/cm^2^ (35% and 49% reduction). As reference, indomethacin (100 μg/cm^2^) and hydrocortisone (20 μg/cm^2^) reduced MPO activity by 36% and 40% after 6 h, or 30% and 46% after 24 h, respectively.

**Table 2:** Topical anti-inflammatory activity of *Rhus coriaria* extracts: effect on ear neutrophilic granulocyte infiltration (MPO activity) induced by Croton oil in mice

Group of treatment	N°. an	N°. an	6 h		24 h	
		Dose (µg/cm^2^)	MPO (EU/mL) Mean ± SEM	Inhibition (%)	MPO (EU/mL) Mean ± SEM	Inhibition (%)
Control	10	–	25.6 ± 1.5	–	62.0 ± 6.3	–
mERC	10	100	23.7 ± 1.1	7	61.2 ± 5.7	1
	10	300	19.7 ± 0.6**	23	51.2 ± 7.9	17
	10	1000	18.1 ± 1.0**	29	37.8 ± 5.0*	39
ARC	10	100	22.8 ± 0.9	11	53.0 ± 5.4	15
	10	300	18.4 ± 0.6**	28	40.6 ± 3.6*	35
	10	1000	16.3 ± 0.4**	36	31.8 ± 4.6*	49
Indomethacin	10	100	16.3 ± 0.6**	36	43.1 ± 3.0*	30
Hydrocortisone	9	20	15.4 ± 0.7**	40	33.3 ± 3.8*	46

*p<0.05, **p<0.001 versus controls at the analysis of variance followed by Dunnett’s test

**Fig. 3:**
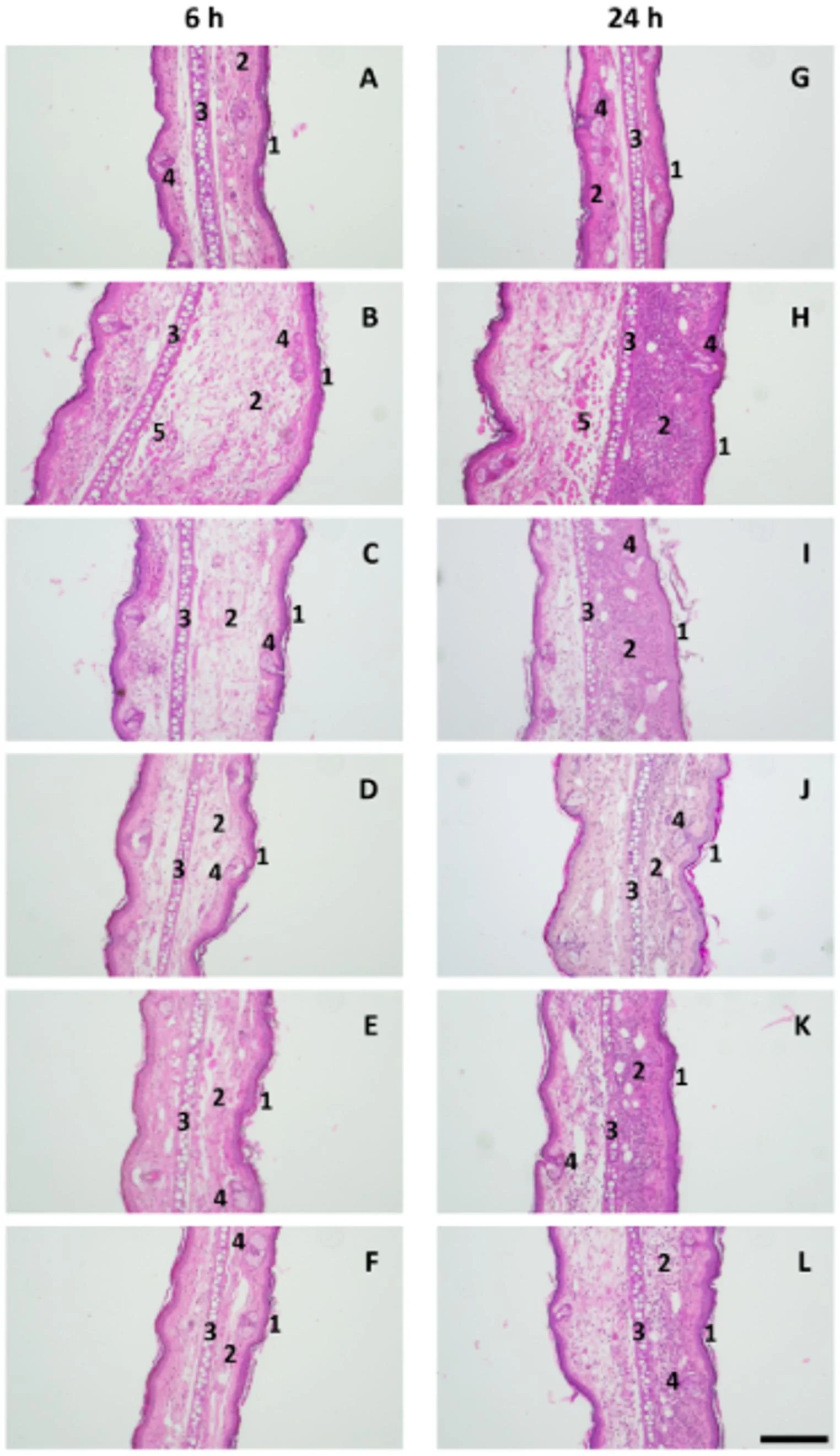
Representative images of transversal sections of auricles from non-treated mice and of mice auricles after dermatitis induction by Croton oil. Samples were collected at 6 h (**A**–**F**) and 24 h (**G**–**L**). No treatment (**A**, **G**), control (**B**, **H**); mERC 1000 μg/cm^2^ (**C**, **I**), ARC 1000 μg/cm^2^ (**D**, **J**); indomethacin 100 μg/cm^2^ (**E**, **K**); hydrocortisone 20 μg/cm^2^ (**F**, **L**). 1: Epidermis; 2: Dermis; 3: Elastic cartilage; 4: Sebaceous gland; 5: Striated muscle. Hematoxylin and eosin staining; original objective magnification: 10 x, scale bar: 200 μm. The inflamed side of the auricle is to the right of cartilage, between cartilage and epidermis

The in vivo effects of *R. coriaria* extracts and of the reference anti-inflammatory drugs were corroborated by the histological analysis of mouse auricles. At 6 h after the dermatitis induction, compared with the auricles of untreated mice (Fig. 3A), auricles from mice treated with Croton oil alone (controls) were characterized by tissue changes mainly associated with edema formation. These included an increased thickness of the dermis due to the tissue swelling, visible on the side of the auricle treated with the irritant (Fig. 3B). These signs of inflammation were attenuated by the topical application of mERC or ARC (100–1000 μg/cm^2^) (Fig. 3C, D), or the reference anti-inflammatory drugs indomethacin (100 μg/cm^2^) or hydrocortisone (20 μg/cm^2^) (Fig. 3E, F). Moreover, as compared to the auricle of untreated mice (Fig. 3G), at 24 h after dermatitis induction, the dermis of control mice was characterized by a marked infiltration of neutrophilic granulocytes (Fig. 3H), which was reduced by the topical treatment with *R. coriaria* extracts (mERC or ARC; 100–1000 μg/cm^2^) (Fig. 3I, J, respectively), indomethacin (100 μg/cm^2^) or hydrocortisone (20 μg/cm^2^) (Fig. 3I, L, respectively).

Our previous in vitro studies on HaCaT skin keratinocytes stimulated with TNF-α (Khalilpour et al. 2019) suggested the involvement of NF-κB in the anti-inflammatory mechanism of sumac fruit extracts. However, in vivo skin exposure to Croton oil induces dermatitis involving the activation of multiple inflammatory pathways and mediators (Mao et al. 2024). Thus, to obtain broader information about the molecular mechanisms involved in the in vivo anti-inflammatory activity of mERC and ARC, mRNA samples from mouse auricles were collected 6 h after dermatitis induction and profiled by RT-PCR array.

**Fig. 4:**
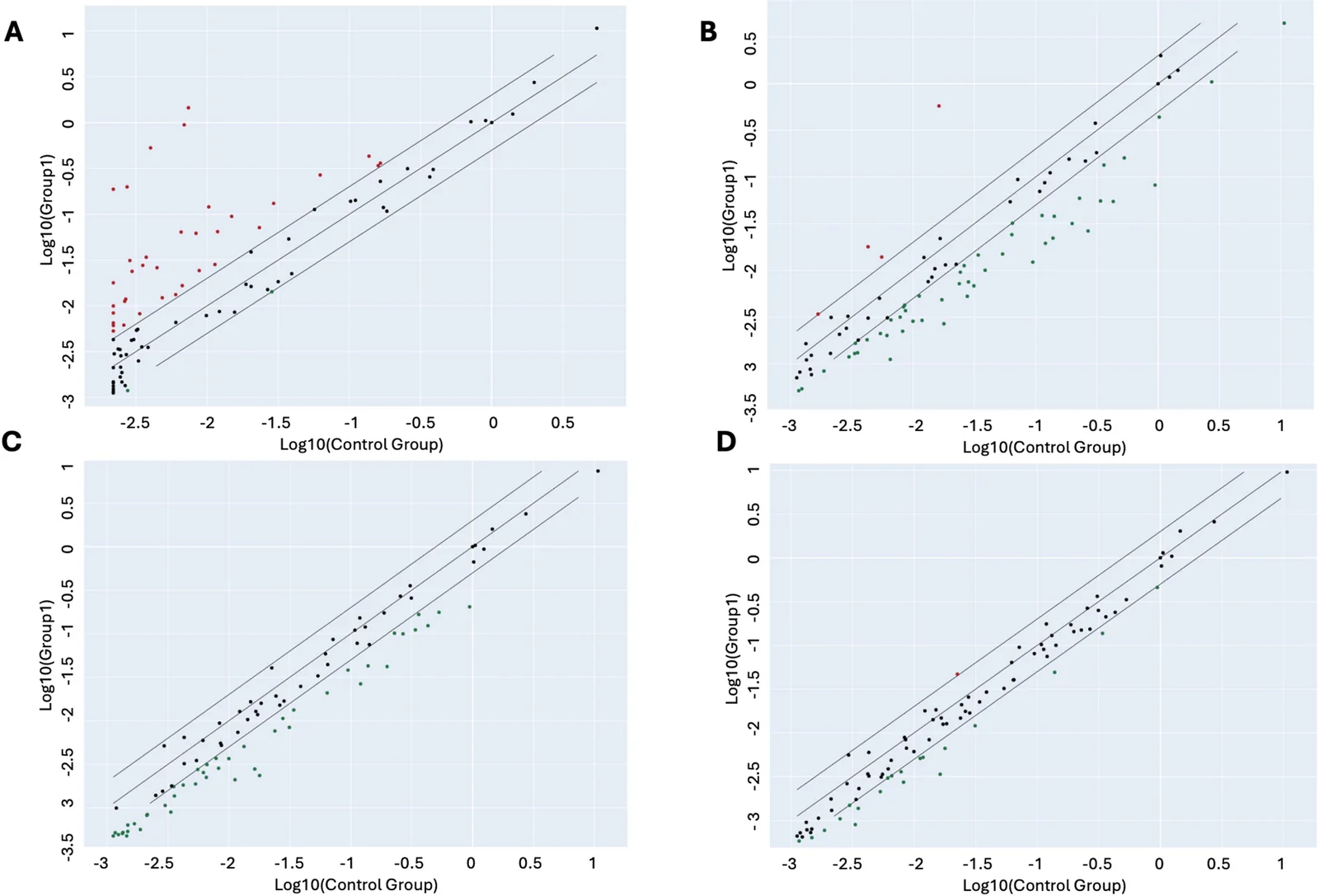
Scatter plots of the expression profile of inflammatory genes. Gene expression was measured by PCR array in mice ear’s tissues after Croton oil application in addition to ARC or mERC (6 h). Plots of unstimulated control (**A**), hydrocortisone (**B**), ARC (**C**), and mERC (**D**) versus Croton oil are reported, respectively. The center diagonal line indicates unchanged gene expression (black dots), while the outer diagonal lines indicate the selected fold regulation threshold. Genes with data points beyond the outer lines in the upper left and lower right corners are up-regulated (red dots) or down-regulated (green dots), respectively, by more than the fold regulation threshold in the y-axis Group relative to the x-axis Group. Scatter plots were automatically generated by GeneGlobe web portal analysis (Qiagen)

**Fig. 5:**
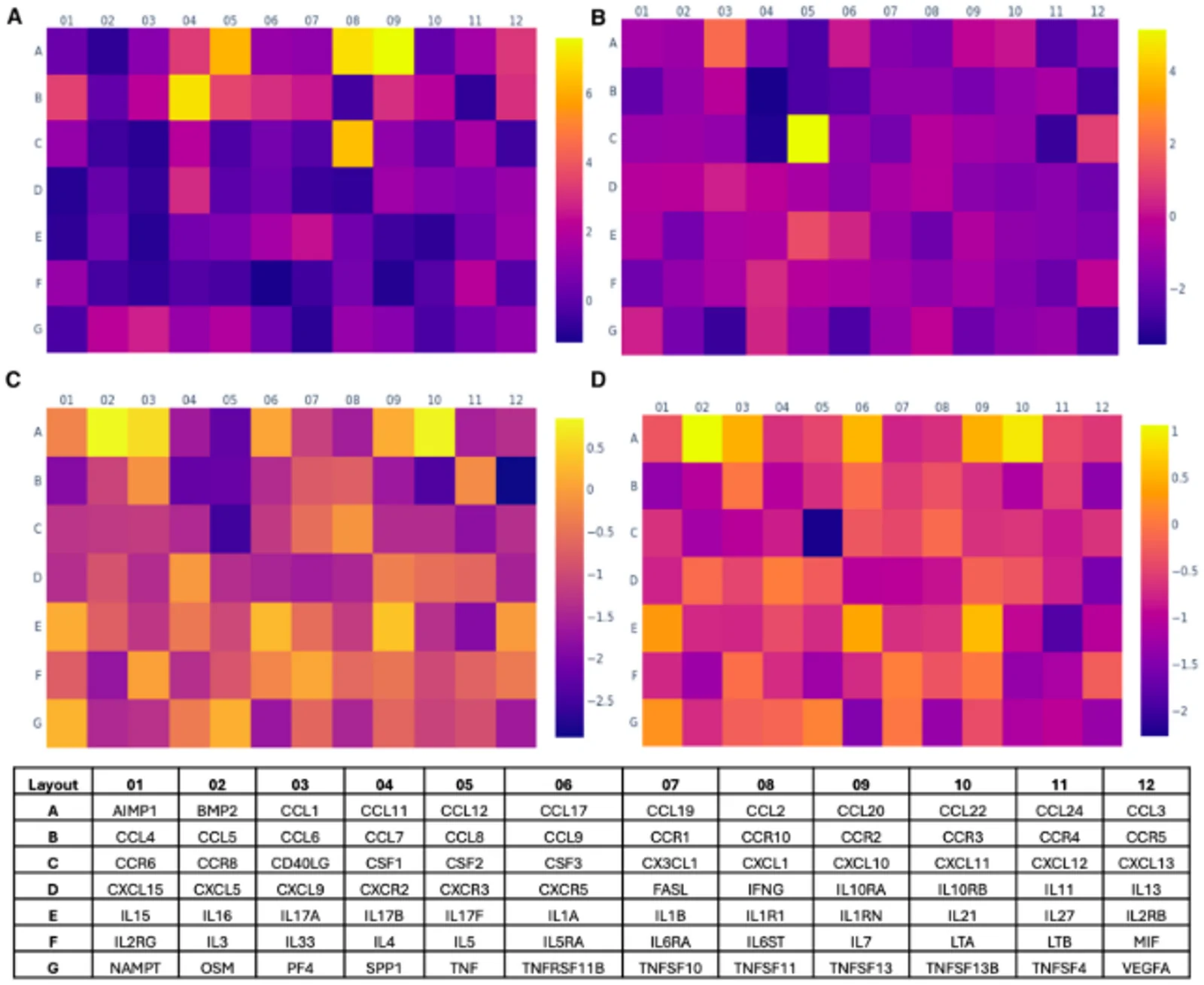
Heat maps of the expression profile of inflammatory genes. Gene expression measured by PCR array in mice ear’s tissues after Croton oil application in addition to ARC or mERC (6 h). Plots of unstimulated control (**A**), hydrocortisone (**B**), ARC (**C**), and mERC (**D**) versus Croton oil are reported, respectively. Heat maps with relative Log scales of fold changes, associated to gradients of color, were automatically generated by GeneGlobe web portal analysis (Qiagen)

As expectable, 36 of 84 genes were over-expressed in Croton oil group, of which the following 17 showed statistical evidence: *CCL11*, *CC17*, *CCL2*, *CCL20*, *CCL4*, *CCL6*, *CCL7*, *CCL8*, *CCR2*, *CSF1*, *CXCL1*, *CXCR2*, *IL13*, *IL2RG, LTB, TNF, TNFSF11, VEGFA*. A summary of gene modulation was described by scatter plots (shown in Fig. 4A) and heatmaps (shown in Fig. 5A), while overall data including fold changes were reported in Supplementary data (Table S1). The relevance and the function of top modulated genes for skin inflammation were also summarized in Table S2.

Conversely, hydrocortisone reversed the inflammatory expression profile by under-regulating 49 genes, of which 15 significantly (shown in Figs. 4B, and 5B). The topical treatment with ARC (shown in Figs. 4C, and 5C) or mERC (shown in Figs. 4D, and 5D) under-regulated a smaller number of genes, likely responsible for the anti-inflammatory effect at phenotypic level. A few of these genes were significantly under-regulated by both extracts, namely *IL13, TNFSF11, TNFSF11B,* and *VEGFA*, while *CCL8*, *CXCL10*, and *IL3* were under-regulated by ARC only. In these regards, fold regulation data with associated *p* values are summarized in Table 3.

**Table 3:** Summary of significantly under-regulated genes after in vivo treatment with ARC or mERC (6 h)

ARC			mERC		
Gene	Fold reg	*p* value	Gene	Fold reg	*p* value
*IL13*	− 2.89	0.011742	*IL13*	− 3.00	0.015102
*TNFSF11*	− 2.84	0.003831	*TNFSF11*	− 2.49	0.043237
*TNFSF11B*	− 3.25	0.009677	*TNFSF11B*	− 2.82	0.021109
*VEGFA*	− 3.07	0.009447	*VEGFA*	− 2.48	0.016823
*CCL8*	− 4.54	0.026812			
*CXCL10*	− 2.63	0.039407			
*IL3*	− 3.33	0.049409			

### In vitro confirmation of the anti-inflammatory targets of sumac extract

The transcription data suggested that both angiogenic (VEGF) and pro-inflammatory (IL13, TNF) pathways were affected by sumac extracts. These data were in part in line with previous experiments in which mERC inhibited the release of several NF-κB-dependent mediators in HaCaT cells stimulated by TNF-α, including VEGF (Khalilpour et al. 2019). Intriguingly, the modulation of the IL13 was a novel finding, thus encouraging the investigation of the IL13 pathway in vitro. IL13 shares a receptor complex with IL4, which is equally crucial in the proallergic response. HaCaT cells can respond to IL4 and IL13 by releasing the STAT6-dependent mediators eotaxins, such as CCL26 (Piazza et al. 2022; Kim et al. 2018) playing a crucial role in eosinophilic infiltration. As a further aspect of interest, ARC was not investigated in human keratinocytes in our previous work (Khalilpour et al. 2019).

For this reason, we decided to compare the bioactivity of mERC and ARC in HaCaT cells stimulated by all the inflammatory effectors identified in the in vivo study, namely the TNF family and IL13. For the purpose, IL8 was selected as NF-κB-dependent chemokine expressed following PMA or TNF induction, while CCL26 was selected as STAT6-dependent chemokine expressed after IL4/IL13 induction (Piazza et al. 2022). Within the TNF family, TNF-α was selected as a representative cytokine to compare the extracts and validate the biological mechanism, in line with our previous work (Khalilpour et al. 2019). As expected, during TNF-α stimulation, mERC and ARC inhibited IL8 release at both time points (shown in Fig. S1). However, IC_50_ values were close to 1.5 µg/mL, thus notably lower than those obtained against PMA stimulation (1.32 μg/mL and 1.63 μg/mL for mERC and ARC, respectively). Once again, mERC was slightly more powerful than ARC, which was therefore selected for the subsequent experiment.

**Fig. 6:**
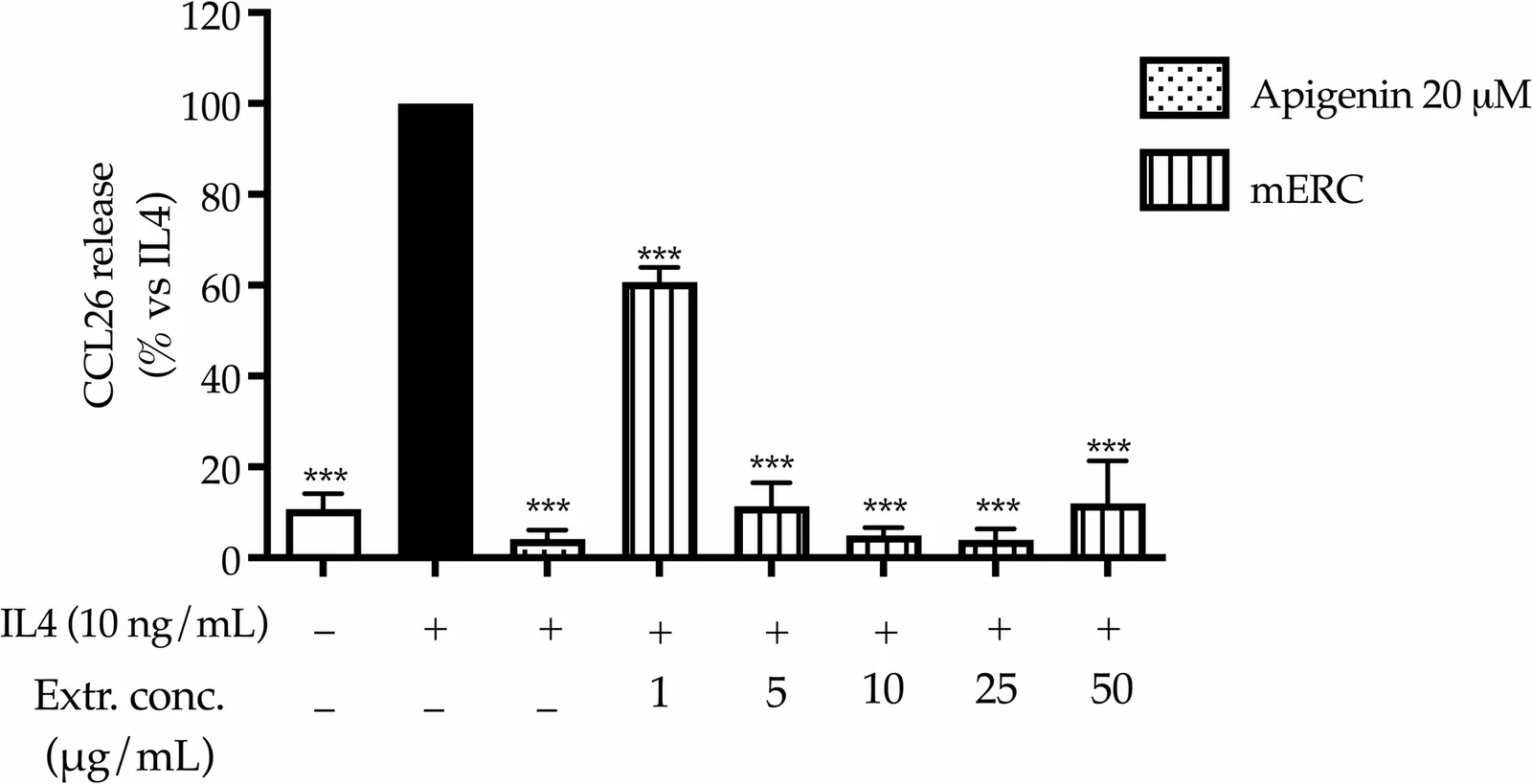
Effect of the treatment with mERC in addition to IL4 on CCL26 release in HaCaT cells (24 h). Calculated IC_50_ was 1.06 μg/mL. The data (ELISA assay) are expressed in percentages, relative to the stimulated control (black column), which is arbitrarily assigned a value of 100%. ARC, acetone extract from *Rhus coriaria* L. fruit, mERC, macerate ethanol extract *Rhus coriaria* L. fruit. ***p < 0.001 (one-way ANOVA) versus stimulus

The last pathway emerged from mRNA data was also evaluated: again, mERC impaired the release of CCL26 induced by IL4 with an IC_50_ of 1.06 µg/mL, with C.I. 95% ranging from 0.56 to 2.01 (shown in Fig. 6), thus confirming the initial hypothesis related to the main mechanism of action.

### LC-HRMS phytochemical profile of sumac extract

**Fig. 7:**
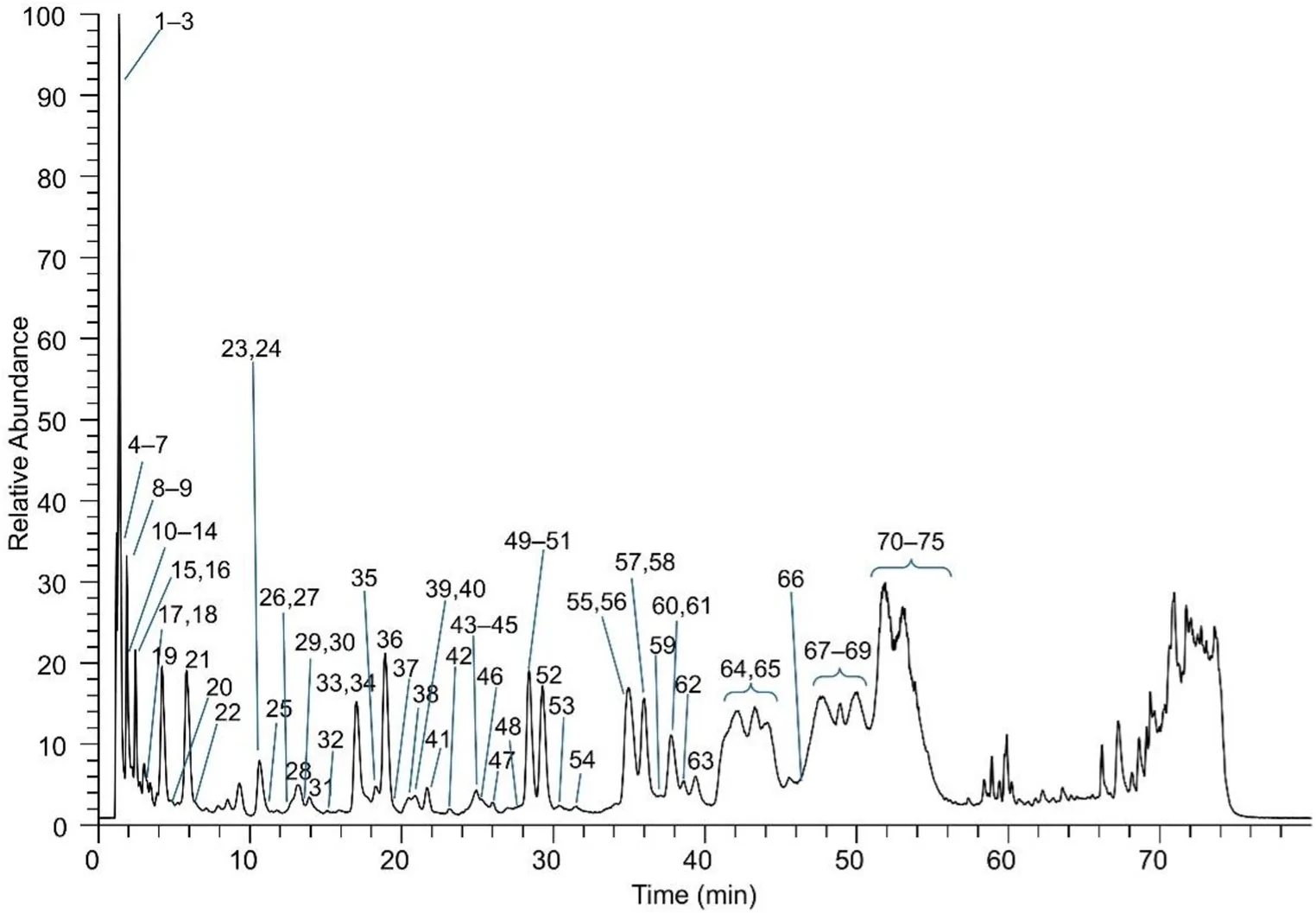
Total ion current chromatogram relative to the LC–MS/MS analysis of sumac extract. Progressive identification number are explained in Table 5

The overall biological experiments guided the selection of mERC for a deep characterization, achieved by targeted LC-HRMS analysis. The data analysis allowed the putative identification of 75 compounds reported in Table 5 and Fig. 7 with the retention time, experimental *m/z* and fragment ions. Among the identified compounds there are malic acid and its glycated form, as well as a lot of molecules bound to malic acid such as gallic acid but also some flavonoids such as chrysoriol-hexose malic acid (isomers I and II) and myricetin-rhamnose malic acid (isomers I and II). A total number of 25 flavonoids were detected in the extract, mainly myricetin, kaempferol and quercetin derivatives. In line with our previous data (Khalilpour et al. 2019), the most abundant class is represented by gallotannins, all characterized by the loss of a gallic acid ([M-H-170]^−^) or galloyl ([M-H-152]^−^) moieties. High molecular weight gallotannins were also detected up to the dodecagalloylhexose derivative.

## Discussion

*Rhus coriaria* L. (sumac) fruit is considered a promising nutraceutical ingredient showing relevant biological properties for human health (Calabro et al. 2023). A recent review article collected the evidence regarding the anti-inflammatory properties of *Rhus* spp. in preclinical studies (Rodriguez-Castillo et al. 2025). Most of the available experimental data have focused on innate inflammatory mediators (TNF, IL-6) involved in cardiometabolic and gastrointestinal disorders. The species *Rhus coriaria* L. has been less investigated compared to other similar species, such as *Rhus verniciflua* Stokes (whose accepted name is *Toxicodendron vernicifluum* (Stokes) F.A.Barkley) and *Rhus chinensis* Mill. Similarly, Bahari et al. have reviewed the anti-inflammatory activity of *Rhus coriaria* L. in preliminary clinical studies conducted in diabetes and overweight patients (Bahari et al. 2024), showing nonuniform results.

Sumac has demonstrated wound healing properties in previous studies, which investigated the topical application of elevated doses of polar fruit extracts (10% ointment and 10 mg/Kg, respectively) (Alsarayreh et al. 2022; Gabr and Alghadir 2019). However, experimental data on skin inflammatory conditions are still lacking. The literature agrees in attributing the biological activities of sumac to polyphenols, mainly represented by gallotannins and flavonoids (Alsamri et al. 2021; Elagbar et al. 2020; Hashem-Dabaghian et al. 2022). Other sources of polyphenols are receiving increasing attention as novel therapeutic approaches to inflammatory skin diseases, due to their antimicrobial, antioxidant, anti-inflammatory, and anti-allergic properties (Salazar et al. 2025).

We previously demonstrated that a macerated ethanolic extract (mERC) exhibits anti-inflammatory activity in TNF-exposed human keratinocytes (HaCaT cells), involving the impairment of the NF-κB pathway (Khalilpour et al. 2019). The bioactivity was clearly correlated with a high polyphenol content, with gallotannins representing the main representative class (Khalilpour et al. 2019). Moreover, an acetone extract (ARC), rich in tannins, showed comparable in vitro bioactivity in a model of *H. pylori* infection (Martinelli et al. 2022), but its effects on skin inflammatory conditions have not yet been investigated. Here, we compared the efficacy of mERC with that of ARC in human skin keratinocytes stimulated with selected pro-inflammatory cytokines and in a mouse model of skin inflammation (Croton oil-induced ear dermatitis).

Both extracts showed no cytotoxicity in HaCaT cells (shown in Fig. 1) and exerted inhibitory activity against PMA-induced release of the pro-inflammatory cytokine IL8 (shown in Fig. 2). These data prompted the investigation of their in vivo topical anti-inflammatory activity using the Croton oil-induced ear dermatitis assay in mice, a standardized model of acute inflammation, widely used for the discovery of new anti-inflammatory agents of plant origin. Croton oil is an irritant containing phorbol esters, mainly PMA, which can activate protein kinase C, triggering multiple downstream pathways that lead to an inflammatory cascade. This cascade includes the activation of phospholipase A2, cyclooxygenase and lipoxygenase pathways, and the production or release of different pro-inflammatory mediators, such as eicosanoids, nitric oxide, cytokines, matrix metalloproteinases and other molecules (Mao et al. 2024; Towbin et al. 1995). The in vivo biological assay showed that mERC and ARC (100–1000 μg/cm^2^) exerted comparable dose-dependent inhibitory effect on edema formation (gravimetric measurement) and neutrophilic granulocytes infiltration (MPO activity) at 6 h and 24 h after dermatitis induction (Tables 1 and 2), which was visible also by the histological analysis of the auricle tissues (shown in Fig. 3). These effects (mERC: up to 31%–41% edema inhibition and 29%-39% MPO activity inhibition at 6 and 24 h, respectively; ARC: up to 46%–55% edema inhibition and 36%-49% MPO activity inhibition at 6 and 24 h, respectively) are comparable to those of raw extracts from other plant species previously shown to possess anti-inflammatory activity by the same experimental model (Sosa et al. 2005; Conforti et al. 2008; Camponogara, et al. 2019). Moreover, mERC and ARC effects were recorded at doses lower than those topically applied by Gabr and colleagues in wound healing experiments (Gabr and Alghadir 2019). Thus, mERC and ARC activity is noteworthy considering that they are crude extracts, which the active constituents are likely diluted by a substantial amount of inert or non-active components. This observation suggests either that the active compounds possess a high intrinsic anti-inflammatory activity or that the observed effect may result from synergistic interactions among multiple constituents present in these extracts. Anyway, the significant in vivo anti-inflammatory effects of the tested extracts suggest their potential application in the treatment of inflammatory skin diseases. However, considering that the Croton oil-induced dermatitis is a model of acute inflammation, which does not reproduce the persistent features of a chronic phlogistic process, further studies are needed to assess the extracts’ ability to attenuate also chronic inflammatory conditions at the skin level.

Transcriptional data, collected by PCR profilers of 84 inflammatory genes, showed that mERC and ARC share a small group of target genes, including IL13, VEGF and TNF-related cytokines (shown in Figs. 4, 5, Table 3). Intriguingly, not only were NF-κB target genes down-regulated, but also other pathways were affected, suggesting the involvement of multiple mechanisms at the transcriptional level. Despite data collected at transcriptional level provided only a partial overview on the mechanism of action which may have occurred during in vivo treatment, in vitro experiments were carried out to investigate the bioactivity of mERC on specific pathways. Thus, the incomplete characterization of the convergent targets affected by the extracts is a limitation of our study.

The two extracts were also compared in HaCaT cells challenged with pro-inflammatory stimuli identified from in vivo data: 1) TNF-α, representative of TNF-related cytokines; 2) IL4, representative of IL13 pathway. IL8 was selected as a well-known NF-κB-dependent mediator expressed after PMA or TNF exposure, while CCL26 was selected as a well-known STAT6-dependent mediator induced by IL4/IL13 exposure (Piazza et al. 2022). Both the extracts inhibited the TNF-α-induced release of IL8, with mERC showing slightly lower IC_50_ (shown in Fig. S1). Consequently, the effect of mERC was also evaluated under IL4 stimulation (shown in Fig. 6). The overall data suggested a more potent inhibitory activity against TNF-α and IL4 (IC_50_ close to 1 μg/mL), compared with PMA induction (IC_50_ close to 20 μg/mL). A summary of the IC_50_ values is reported in Table 4. Intriguingly, other publications, including from our group (Piazza et al. 2022), correlated the anti-allergic and anti-inflammatory properties of plant extracts with the presence of gallotannins as characterizing polyphenols (Choi et al. 2021; Lee et al. 2019).

**Table 4:** IC_50_ of mERC related to inflammatory markers released by human HaCaT keratinocytes exposed to TNF-α, PMA or IL4 for 24 h

Stimulus	Marker	IC_50_ (μg/mL)	I.C. (95%)
TNF-α	IL8	1.32	0.8574 to 2.023
PMA	IL8	18.68	10.97 to 31.82
IL4	CCL26	1.06	0.5566 to 2.013

To further support this hypothesis, an in-depth characterization of mERC was performed (Table 5), thus confirming the presence of gallotannins of various molecular weights. Distinct polyphenols bound to malic acid—including gallic acid, flavonols (e..g, myricetin, kaempferol, and quercetin), and flavones (e.g., chrysoriol)—were also tentatively identified. Our data are fully consistent with previous studies in which hydroalcoholic extracts from sumac fruit were analyzed (Abu-Reidah et al. 2015; Grassia et al. 2021; Mazzara et al. 2023; Tohma et al. 2019; Kosar et al. 2007; Scarano et al. 2024; Shabana et al. 2011).

**Table 5:** Compounds identified by LC-HRMS in *Rhus coriaria* L. (mERC extract)*,* the calculated and experimental m/z (with the mass tolerance reported in ppm), the MS/MS fragments, the retention time (RT, minutes), and the identification number (N.) relative to Figure X

N	Compound	Calc *m/z* [M–H]^−^	Calc *m/z* [M–2H]^2−^	RT (min)	Experimental *m/z*	MS/MS	Δ ppm
1	Malic acid hexoside	295.0671		1.4	295.0672	179–133	0.44
2	Malic acid	133.0142		1.4	133.015	–	0.001
3	Dimaloyl dihexoside	573.1309		1.4	573.1323	341–457	2.526
4	Dimalic acid	249.0252		1.5	249.026	133	3.191
5	Galloylhexose IV	331.0671		1.5	331.0676	169	1.691
6	Galloylhexose malic acid I	447.078		1.5	447.0791	169–331	2.463
7	Digalloyl-hexoside I	483.078		1.5	483.0794	169–331	2.921
8	Galloylhexose I	331.0671		1.8	331.0665	169	− 1.722
9	Galloylhexose malic acid II	447.078		1.8	447.0788	169–331	1.792
10	Digalloyl-hexoside II	483.078		1.9	483.0788	169–331	1.658
11	Gallic acid	169.0143		2	169.015	125	4.16
12	Galloylhexose II	331.0671		2.1	331.0664	169	− 1.904
13	Galloyl shikimic acid i	325.0565		2.2	325.0559	169–173	− 1.924
14	Digalloyl maloyl hexose	599.089		2.2	599.0884	169–331–483	− 1.014
15	Maloyl gallic acid	285.0252		2.4	285.0256	169–133	1.384
16	Galloylhexose III	331.0671		2.6	331.0669	169	− 0.514
17	Dihydroxybenzoic acid	153.0193		3.2	153.0197	109	2.208
18	Galloyl quinic acid	343.0671		3.3	343.0672	169–191	0.291
19	Tri-galloyl-hexoside I	635.089		4.2	635.0874	331–483	− 2.5
20	Coumaryl-hexoside	325.0929		5.2	325.0927	163	− 0.649
21	Tri-galloyl-hexoside II	635.089		5.9	635.0881	331–483	− 1.397
22	Trigalloyl maloyl hexose	751.1		6.1	751.0999	331–483–635	− 0.061
23	Trigalloyllevoglucosan I	617.0784		10.8	617.0789	295–313–447–465	0.806
24	Tetragalloylhexose I	787.1		10.8	787.0965	483–635	− 4.378
25	Chrysoriol-hexose malic acid I	577.1199		11.1	577.1198	299–461	− 0.145
26	Diosmetin derivative	613.1199		12.6	613.1195	285–299	− 0.642
27	Myricetin galloyl hexoside	631.1305		12.6	631.1305	317–479	0.066
28	Myricitin derivative	515.0467		13.1	515.0446	317–339	− 4.133
29	Chrysoriol-hexose malic acid II	577.1199		13.3	577.1193	299–461	− 0.994
30	Myricetin O-rhamnosylglucose	625.141		13.3	625.14	317–479	− 1.636
31	Myricetin hexoside	479.0831		13.8	479.0836	317	0.993
32	Myricetin hexosyl hexuronide	655.1152		15	655.1157	317–493	0.699
33	Trigalloyllevoglucosan II	617.0784		17	617.0781	295–313–447–465	− 0.588
34	Tetragalloylhexose II	787.1		17	787.0961	483–635	− 4.924
35	Ellagic acid	300.999		18.4	300.9989	185–229–257–273	− 0.401
36	Myricetin-3-O-rhamnoside	463.0882		19	463.0883	317	0.26
37	Eriodictyol hexoside or Dihydrokaempferol hexoside	449.1089		19.5	449.1093	287	0.768
38	Quercetin rutinoside	609.1461		20.4	609.1464	301	0.479
39	Quercetin glucuronide	477.0675		20.8	477.0677	301	0.536
40	Trigalloyllevoglucosan III	617.0784		20.8	617.0784	295–313–447–465	− 0.086
41	Quercetin hexoside	463.0882		21.7	463.0891	301	1.901
42	Kaempferol hexoside	447.0933		23	447.0935	285	0.57
43	Myricetin-rhamnose malic acid I	579.0992		24.8	579.0991	317–463	− 0.066
44	Trigalloyllevoglucosan IV	617.0784		24.8	617.0785	295–313–447–465	0.109
45	Pentagalloyl-hexoside I	939.1109		24.9	939.1106	635–787	− 0.324
46	Quercetin arabinoside	433.0776		25.3	433.0776	301	0.012
47	Myricetin-rhamnose malic acid II	579.0992		26	579.0994	317–463	0.469
48	Kaempferol rutinoside	593.1512		27.8	593.1514	285	0.297
49	Trigalloyllevoglucosan V	617.0784		28.3	617.0782	295–313–447–465	− 0.377
50	Di-O-galloyl-2,3-(S)-hexahydroxydiphenoyl-scyllo-quercitol II	769.0894		28.3	769.0894	465–617	0.024
51	Pentagalloyl-hexoside II	939.1109		28.3	939.1106	447–617–769	− 0.324
52	Quercetin rhamnoside	447.0933		29.3	447.0937	301	0.973
53	Myricetin	317.0303		30.3	317.0302	137–151–179–241	− 0.35
54	Apigenin glucoside I	431.0984		31.4	431.0982	269	− 0.442
55	Trigalloyllevoglucosan VI	617.0784		34.8	617.0784	295–313–447–465	− 0.086
56	Hexagalloylhexose I	1091.122		34.8	1091.123	769–939	0.95
57	1,5-di-O-galloyl-3,4-(S)-hexahydroxydiphenoyl protoquercitol	769.0894		35.9	769.0895	465–617	0.128
58	Hexagalloylhexoside II	1091.122		35.9	1091.121	939	− 0.425
59	Quercetin-rhamnose malic acid	563.1042		36.9	563.1047	301–447	0.882
60	Trigalloyllevoglucosan VII	617.0784		37.9	617.0783	295–313–447–465	− 0.28
61	Hexagalloyl-hexoside III	1091.122		37.9	1091.122	769–939	0.034
62	Kaempherol rhamnoside	431.0984		38.5	431.0988	285	1.043
63	Hexagalloyl-hexoside IV	1091.122		39.2	1091.123	939	0.675
64	Heptagalloylhexose I	1243.133	621.0628	42	621.0626	469–545	− 0.171
65	Heptagalloylhexose II	1243.133	621.0628	43.2	621.0615	469–545	− 1.008
66	Quercetin	301.0354		46.7	301.0353	151–179–257–273	− 0.419
67	Octogalloylhexose I	1396.149	697.0674	47.6	697.0667	469–545–621	− 0.477
68	Quercetin galloyl deoxyhexoside	599.1042		48.9	599.1047	301–447	0.829
69	Octogalloylhexose II	1396.149	697.0674	50.1	697.0673	469–545–621	− 0.04
70	Octogalloylhexose III	1396.149	697.0674	51.5	697.0656	469–545–621	− 1.307
71	Nonagalloylhexose	1548.159	773.072	52	773.0714	469–545–621–697	− 0.406
72	Decagalloylhexose	1700.168	849.0766	53.3	849.0792	545–621–697–773	1.517
73	Undecagalloylhexose	1852.177	925.0812	53.9	925.0844	545–773	1.71
74	Dodecagalloylhexose	2004.186	1001.086	54.6	1001.091	849	2.542
75	Kaempferol	285.0405		55.6	285.0408	151	1.187

Nevertheless, considering the broad inflammatory action of PMA, further mechanistic studies in models of autoimmune and Th2-driven inflammation could be explored to validate our hypothesis, such as oxazolone and ovalbumin-induced dermatitis. Moreover, the attribution of anti-inflammatory mechanisms to specific polyphenols still deserves a dedicated analysis, including plasma markers and immunological profiling.

Finally, in vitro results corroborated the mechanism of action inferred from in vivo experiments. The results suggest further investigations aimed at addressing the potential role of sumac in allergic or autoimmune skin diseases, such as contact dermatitis, atopic dermatitis, and psoriasis. Accordingly, previous studies have suggested that formulation strategies, such as ethosomes and phytosomes (Emanet et al. 2025) may improve the delivery of polyphenols from sumac, but their role in dermatological therapies remains unexplored. Thus, the impact of these formulations on the anti-inflammatory dose and the safety of sumac extracts after topical application warrants specific investigations.

## Conclusions

The present work demonstrated the efficacy of *Rhus coriaria* fruit extracts against skin inflammation by in vitro and in vivo models. The findings suggested that their mechanism of action may involve the inhibition of TNF-α and IL13 pathways, which play a crucial role in leukocytes recruitment in acute and chronic inflammation. Therefore, our results pave the way for future investigations aimed at elucidating the effect of *Rhus coriaria* extracts towards chronic skin inflammation as well as allergic and autoimmune skin disorders.

## Supplementary Information

Below is the link to the electronic supplementary material.

Supplementary Material 1

## Data Availability

All data generated or analyzed during this study are included in this article and its supplementary material files. Further enquiries can be directed to the corresponding author.
