# Supplementary material for: In vivo and in vitro investigation of skin anti-inflammatory effect of Sumac (Rhus coriaria L.)
Source: Inflammopharmacology. 2026 Aug 26;34(9):6119–34. doi: 10.1007/s10787-026-02368-2 (PMC13558293; doi:10.1007/s10787-026-02368-2)
Supplement: Supplementary file 1 [file 10787_2026_2368_MOESM1_ESM.docx]

**Supplementary data**

**Table S1.** Fold changes with respective *p* values of the expression profile of inflammatory genes measured by PCR array in mice ear’s tissues after Croton oil application (6 h). The effect of Croton oil is described at left, while the effect of concomitant treatment with hydrocortisone is described at right. Tables and *p* values were automatically generated by GeneGlobe web portal analysis.

| **Position** | **Gene Symbol** | **Fold Regulation** | **p-Value** |
| --- | --- | --- | --- |
| A03 | *CCL1* | 4.18 | 0.040342 |
| A04 | *CCL11* | 9.73 | 0.027294 |
| A06 | *CCL17* | 2.53 | 0.032336 |
| A08 | *CCL2* | 132.08 | 0.034531 |
| A09 | *CCL20* | 195.99 | 0.002620 |
| B01 | *CCL4* | 10.90 | 0.048166 |
| B03 | *CCL6* | 4.47 | 0.029822 |
| B04 | *CCL7* | 136.67 | 0.033828 |
| B05 | *CCL8* | 11.70 | 0.011310 |
| B05 | *CCL8* | -6.17 | 0.028787 |
| B09 | *CCR2* | 8.01 | 0.012155 |
| B09 | *CCR2* | -3.31 | 0.048517 |
| C04 | *CSF1* | 4.30 | 0.032457 |
| C04 | *CSF1* | -10.17 | 0.035588 |
| C05 | *CSF2* | 35.51 | 0.003320 |
| C08 | *CXCL1* | 85.28 | 0.009744 |
| D04 | *CXCR2* | 7.40 | 0.008204 |
| D10 | *IL10RB* | -2.91 | 0.027320 |
| D12 | *IL13* | 2.44 | 0.032144 |
| D12 | *IL13* | -3.69 | 0.010896 |
| F01 | *IL2RG* | 2.49 | 0.039809 |
| F01 | *IL2RG* | -3.77 | 0.022798 |
| F08 | *IL6ST* | -2.33 | 0.019411 |
| F09 | *IL7* | -2.00 | 0.033146 |
| F11 | *LTB* | 4.41 | 0.029161 |
| G05 | *TNF* | 3.80 | 0.003309 |
| G05 | *TNF* | -2.05 | 0.048124 |
| G06 | *TNFRSF11B* | -6.25 | 0.008410 |
| G07 | *TNFSF10* | -2.01 | 0.042467 |
| G08 | *TNFSF11* | 2.42 | 0.003511 |
| G09 | *TNFSF13* | -3.86 | 0.040063 |
| G12 | *VEGFA* | 2.13 | 0.023614 |
| G12 | *VEGFA* | -6.12 | 0.007238 |
| H01 | *ACTB* | -2.40 | 0.026498 |
| H04 | *GUSB* | -3.74 | 0.028848 |
| H05 | *HSP90AB1* | -2.62 | 0.017411 |

**Table S2.** Summary and description of the top upregulated genes by PCR array in mice ear’s tissues after Croton oil application (6 h).

| **Gene** | **Role of the respecting protein in skin inflammation** |
| --- | --- |
| *CCL11* | Eotaxin-1 is a chemoattractant for eosinophils in chronic inflammatory and allergic conditions |
| *CCL17* | TARC is a chemoattractant for T lymphocytes with role in autoimmune in allergic conditions |
| *CCL2* | MCP-1 is a chemoattractant for macrophages with role in chronic inflammation |
| *CCL20* | MIP-3α is a chemoattractant for T lymphocytes including Th17 cells |
| *CCL7* | MCP-1 is a chemoattractant for macrophages with role in chronic inflammation |
| *CCR2* | CCR2 is a receptor for chemokines like CCL2, CCL7, CCL8, CCL12, and CCL13 |
| *CSF1* | CSF-1 is a growth factor crucial for the development and function of macrophages |
| *CXCL1* | GRO-α is a chemoattractant for neutrophils |
| *CXCR2* | CXCR2 is a receptor for chemokines like CXCL1 and CXCL8 |
| *IL13* | IL13 is a Th2 cell-derived cytokine with key role in allergic diseases |
| *LTB* | LTB belongs to TNF family |
| *TNF* | TNF promotes innate and autoimmune inflammation |
| *TNFSF11* | RANKL is a cytokine belonging to TNF family |
| *VEGFA* | VEGF-A is a growth factor with a key role in angiogenesis |

**Figure S1.** Effect of the treatment with mERC or ARC in addition to TNF on IL8 release in HaCaT cells at 6 h (A) or 24 h (B). Calculated IC_50_ at 24 h were: 1.63 μg/mL for ARC, and 1.32 μg/mL for mERC, respectively. The data (ELISA assay) are expressed in percentages, relative to the stimulated control (black column), which is arbitrarily assigned a value of 100%. ARC, acetone extract from *Rhus coriaria* L. fruit, mERC, macerate ethanol extract *Rhus coriaria* L. fruit. EGCG, epigallocatechin gallate (20 μM). *p < 0.05, *** p < 0.001 (one-way ANOVA) versus stimulus.
